# RASA4 inhibits the HIFα signaling pathway to suppress proliferation of cervical cancer cells

**DOI:** 10.1080/21655979.2021.2002499

**Published:** 2021-12-06

**Authors:** Junying Chen, Jinbing Huang, Qiaoqiao Huang, Ji Li, Erling Chen, Wensheng Xu

**Affiliations:** a Department of Gynecology, The First Affiliated Hospital of Guangxi Medical University, Nanning City, China; b Department of Obstetrics and Gynecology, The People’s Hospital of Cenxi City, Cenxi city, China

**Keywords:** RASA4, proliferation, HIFα, cervical squamous cell carcinoma

## Abstract

RAS p21 protein activator 4 (RASA4) has been recognized as a Ca^2+^-promoted Ras–MAPK pathway suppressor that inhibits tumor growth. However, the role of RASA4 in cervical squamous cell carcinoma (CESC) remains unclear. The mRNA levels of RASA4 were analyzed using the GEO and GEPIA databases. Kaplan–Meier analysis and ROC analyses were conducted to determine the prognostic and diagnostic values for patients from the TCGA-CSCE cohort. The CCK8 and colony assays were performed to assess the impact of RASA4 ectopic expression and gene inactivation on tumor cell proliferation. In vivo experiments were performed. Luciferase reporter assays and LW6 (a HIFα inhibitor) were employed to verify the regulatory relationship between RASA4 and the HIFa signaling pathway. The GEPIA and GEO database analysis demonstrated poorly expressed RASA4 in the CESC tissues relative to that in the noncancerous tissues. Based on the TCGA database, poorly expressed RASA4 signified high prognostic and diagnostic values. Ectopically expressed RASA4 weakened the proliferative potential of HeLa cells, whereas RASA4 genetic inactivation produced the opposite impact in the HeLa and C-33A cells. The promoting effect of RASA4 deficiency on tumourigenesis was also recorded *in vivo*. Subsequently, RASA4 negatively regulated the HIFα-driven luciferase activities and weakened the expression of survivin. Meanwhile, LW6 treatment abrogated the increased proliferation of HeLa cells, as well as the increased expression of survivin by RASA4 depletion. Our findings indicated that RASA4 can inhibit the proliferation of cervical cancer cells by inactivating the HIFα signaling pathway, suggesting novel prospects for targeted therapy against CESC.

## Introduction

Cervical cancer (CC) is a malignant disorder of the uterine cervix. In 2020, approximately 604,127 new cases of CC and 341,831 new deaths from CC were recorded worldwide [1]. Advanced chemotherapy regimens have limited therapeutic response rates and grim clinical outcomes [2]. Currently, increasing investigations have proven that molecular targeted therapy can be a promising strategy against CC in addition to immune therapy [3,4]. Therefore, a deeper understanding of the molecular mechanism behind CC progression is highly required.

As a dominant member of the GTPases group, the RAS proteins act as molecular switches to control the mitogen-activated protein kinase (MAPK) cascade. Its aberrant expression has been evidenced as a biomarker of various human cancers [5,6]. The activation of mutations in the RAS genes frequently occurs in CC owing to its influence on cellular growth and differentiation, which causes malignant transformation and aids cancer progression [7,8]. RAS p21 protein activator-4 (RASA4), also called GAPL and CAPRI, is a calcium-dependent GTPase-activating protein that facilitates conversion from the active GTP-bound state to the inactive GDP-bound state. This conversion causes the deactivation of the RAS gene, thereby inhibiting tumor progression. The genetic loss of the RASA family is well-recognized in different malignancies. On the other hand, this protein is calcium-dependent. Upon activation by intracellular Ca(2+), this protein gets translocated to the plasma membrane, attributing to the RAS inactive status.

When exposed to the intracellular Ca(2+) overload condition, RASA4 can cause the conversion of Ras to the inactive status, resulting in the inactivation of Ras signaling events, which plays an important role in controlling the cell fate and inhibiting cancer progression. Studies have demonstrated that RASA4 deregulation in cancer contributes to RAS inactivation, thereby inhibiting the MAPK cascade [9,10]. Another prominent example of RASA4 is that RASA4 hypermethylation causes its downregulation in resistant juvenile myelomonocytic leukemia, which is associated with a grim prognosis [11]. However, only little is known about the expression status and role of RASA4 in CC progression.

Hypoxia has a considerable influence on the progression of a myriad of human malignancies [12]. Hypoxia-inducible factor-1α (HIF-1 α) acts as a crucial modulator of the cellular and systemic homeostatic responses to hypoxia by orchestrating the transcription of diverse genes. These HIF-target genes exhibit propounding correlation with diverse aspects of tumourigenesis, such as proliferation, metabolism, and immune escape [12]. On the other hand, increased stimuli from mitochondrial Ca(2+) can trigger the activation of HIF1α signaling to confer metastatic properties to human colorectal tumor cells.

Considering the connection of Ca(2+) with RASA4 and HIF1α, we hypothesized that RASA4 may negatively regulate HIF1α signaling and suppress tumor growth. In the present study, we attempted to uncover the functions and the underlying regulatory mechanisms of RASA4 in CC. Our findings substantiated that RASA4 suppresses the proliferation of HeLa and C-33A cells by inactivating the HIF1α signaling pathway, indicating that RASA4 may be a promising candidate against CC.

## Materials and methods

### Cell culture, plasmids, and antibodies

The human CC cell lines HeLa and C-33A were purchased from China Center for Type Culture Collection (Wuhan, China). All the cells were cultured in Eagle’s Minimum Essential Medium supplemented with 10% FBS and incubated at 37°C under a 5% CO_2_ atmosphere. The HRE-luciferase reporter construct was purchased from addgene for the evaluation of the HIF1α transcriptional activity. The pLV-Flag vector was obtained commercially from Inovogen Tech (USA). Human anti-RASA4 antibodies (ab151232, 1:1000), anti-flag antibodies (ab93713, 1:1000), anti-GAPDH antibodies (ab8245, 1:1000), and anti-survivin antibodies (ab76424, 1:1000) for Western blotting were purchased from Abcam (Cambridge, MA, USA).

### Transcript expression, prognosis, and diagnostic value analyses

The differential levels of RASA4 between the CESC tissues and normal cervical tissues were analyzed using the GEO database and GEPIA platform, as described in a study [13].

Furthermore, the RASA4 expression data were obtained from the TCGA-CESC cohort. Kaplan–Meier analysis was conducted to analyze the overall survival of RASA4-high and RASA4-low patients. In addition, a received operating characteristic (ROC) curve was generated to determine the diagnostic value of RASA4 expression.

### Establishment of RASA4-overexpressing HeLa cells

RASA4 cDNA was purchased from Wuhan Tianyi Huiyuan Biotechnology Co. Ltd (Wuhan, China) and ligated into pLV-Flag vectors (Invogen Tech. Co., Ltd., Chongqing, China). The recombined vectors pLV-Flag-RASA4 and pLV-Flag were delivered into the HEK293T cells with pLV-Flag backbone vectors by using the Lipofectamine 2000 Reagent (Invitrogen, CA, USA), as per the manufacturer’s protocol. The lentivirus particles were concentrated by filtering through a 0.45-μm sterile filter and enriched through centrifugation for the next infection with HeLa cells by using polybrene (Millipore, USA). After 48 h of incubation, the infected HeLa cells were selected in puromycin-containing culture media for 10 days. The puromycin-resistant colonies were verified through Western blotting and prepared for the subsequent assays.

### Establishment of RASA4-knockout HeLa and C-33A cells

The two sgRNAs targeted RASA4 Exon1 and Exon3 were designed using the sgRNA design tool (http://crispr.dfci.harvard.edu). The sgRNA duplexes (CGAGATCCAC CTG) were generated by Wuhan Tianyi Huiyuan Biotechnology Co., Ltd. and inserted into the lentiCRISPR v2 vector (Addgene #52961). The resultant lentiCRISPR v2-RASA4 vectors and the empty lentiCRISPR v2 vectors were injected into the HEK293T cells with 3-μg psPAX2 packaging plasmid and 1-μg PMD2.G envelope plasmid. The transfection of HeLa and C-33A, as well as the puromycin selection were performed, as described earlier. Finally, the monoclonal cells were propagated and verified through Sanger sequencing and Western blotting. The two knockout clones KO-1 and KO-2 were prepared for the subsequent assays.

### RT-qPCR

Isolation of total RNA was performed using the RNAstorm RNA Isolation Kit (Biotium, USA). Sequentially, cDNA was prepared using the iScript cDNA Kit (BioRad). Specific mRNA amplification and quantification were performed using the Quanti-Tect SYBR Green PCR Kit (QIAGEN, China) with specific primers: RASA4: 5ʹGGATCTGGCCTGCCTTTCTT3ʹ, 5ʹAACATCTGGAGGGCTGGGA3ʹ; survivin: 5ʹTTGCAGCCAATCAAAACCCG3ʹ, 5ʹTAAATCCGCAGGAGTCTCGC3ʹ.

Fold changes were computed using the 2^−ΔΔCT^ method.

### Western blotting

Proteins were isolated from the cells b using the RIPA lysis buffer (Abcam) before quantification with the BCA Kit ((Pierce). The 20-μg proteins were loaded into every slot of the gel and run at 200 V for 1 h. The protein was transferred to a nitrocellulose membrane at 15 V per gel and 4°C for 24 h. After rinsing the membrane with PBS, it was incubated with a 10-mL blocking solution at room temperature for 1 h. Anti-flag antibodies (Cat#: SAB4301135, 1:1000, Millipore Sigma, USA), anti-RASA4 antibodies (Cat#:SAB4301585, 1:1000, Millipore Sigma), anti-survivin antibodies (Cat#:SAB5700745,1:1000, Millipore Sigma), and anti-GAPDH antibodies (Cat#:SAB2108668, 1:1000, Millipore Sigma) were added for another 3 h, following 1 h of incubation with anti-rabbit IgG secondary antibodies (Cat#:AP510 or Cat#:AP120R, 1:1000, Millipore Sigma). Immunoreactive bands were visualized using an ECL solution and captured on X-ray films.

### In vitro proliferation assay

The proliferation was assayed using the CCK8 courting kit (Abcam) according to the manufacturer’s suggestions. Briefly, the cells (1 × 10^3^ cells/well) were seeded into a 96-well plate. After the cells were cultured for 1, 3, and 5 days, 10 uL of the CCK8 solution (Sigma, USA) was supplemented. The optical density (OD) was determined calorimetrically (OD: 450 nm) using an ELISA plate reader (Biotek, Winooski, VT, USA).

### In vitro clonogenic assay

Cells (500 cells/well) were maintained in a 6-well plate and incubated in a CO_2_ incubator at 37°C for 14 days. After rinsing with PBS, the cells were treated with 2–3 mL of the fixation solution for 5 min at room temperature. Following fixation, the cells were stained with 0.5% of the crystal violet solution for 2 h. Finally, the staining was observed through stereomicroscopy (Thermo Fisher Scientific, USA), and the number of colonies (with ≥50 cells per colony) was counted.

LW6 treatment

To ascertain the critical role of HIF1α in RASA4-mediated progress, in some assays, the RASA4-deficient C-33A cells were exposed to 1-μM LW6 (Selleck, USA) for 12 h. The control cells were sham-treated with DMSO (Sigma-Aldrich).

### Dual-luciferase reporter assay

To examine the alternation of the HIF-1 activity in response to RASA4 overexpression, 1 × 10^6^ HeLa cells at 50% sub-confluency were seeded into a 24-well plate for 24 h. The pLV-Flag-RASA4 vectors (0, 50, 100, 200 ng) were introduced into HeLa cells in combination with 20-ng HRE-luciferase reporter construct and 10-ng pRL-SV40-Renilla luciferase. The luminescence signals were read using the dual-luciferase assay system (Promega). The HIF-1 transcriptional activity is presented as the ratio of Firefly/Renilla intensity.

### In vivo experiments

A total of 12 pathogen-free female C57BL/6 mice (age: 6 weeks; weight: about 250 g) were purchased from the Experimental Animal Center of Wuhan University (Wuhan, China). The mice were categorized into two groups and kept in ventilated cages that were illuminated with a 12:12-h light/dark cycle. The animal work was conducted with approval from the Animal Ethics Committee of The Animal Care & Welfare Committee of Guangxi Medical University, China. After 7 days of adaptation, 1 × 10^5^ cells/100 µL of RASA4-deficient C-33A cells (Knockout, KO) or C-33A cells with no treatment (Wild-type cells, WT) were subcutaneously administered into the two groups of mice. After 7 days of subcutaneous injection, the tumor volume (tumor volume = 0.5 × D × d^2^) was detected every 3 days. After 16 days, the xenograft tumors were resected under anesthesia and the tumor weights were recorded.

### Statistical analysis

Triple independent repeats were conducted. The experimental results are presented as mean ± SEM and analyzed using Prism 8 statistical software. The Student’s *t*-test was applied to determine the statistical difference between the two groups. One-way ANOVA, followed by post-hoc test, was adopted for difference comparison among the multiple groups. A P value < 0.05 was considered to indicate statistical significance.

## Results

### RASA4 downregulation in the CESC tissues

**Figure 1.**
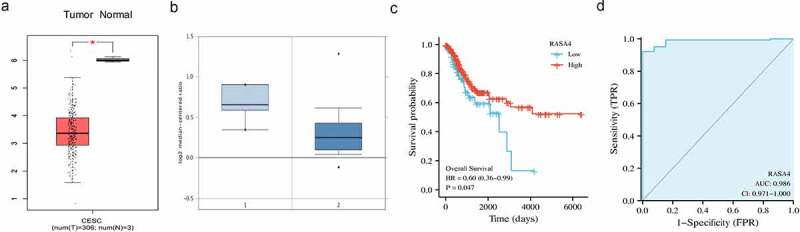
RASA4 downregulation in the CESC tissues. (a) The RASA4 mRNA levels in normal and neoplastic cervical tissues in the GEPIA database. (b) The RASA4 mRNA levels in normal and neoplastic cervical tissues in oncomine databases. (c) Kaplan–Meier curves for overall survival in CESC. (d) The ROC curve of RASA4 to assess the diagnostic value

Through the GEPIA database, significantly reduced RASA4 mRNA levels were detected in the CESC tissues in comparison with those in the normal cervical tissues (Figure 1(a)). Using the data from the GEO database, the differentially decreased expression in the CESC tissues was also recorded (Figure 1(b)). Furthermore, the low-RASA4 expression was associated with a short overall survival rate (Figure 1(c)). In addition, the diagnostic value of RASA4 was assessed using the ROC curve. The results revealed that RASA4 had a good diagnostic value with AUC = 0.986 (Figure 1(d)). All data underscored the involvement of RASA4 in CESC progression.

### RASA4 overexpression suppresses proliferation and colony formation in HeLa cells

**Figure 2.**
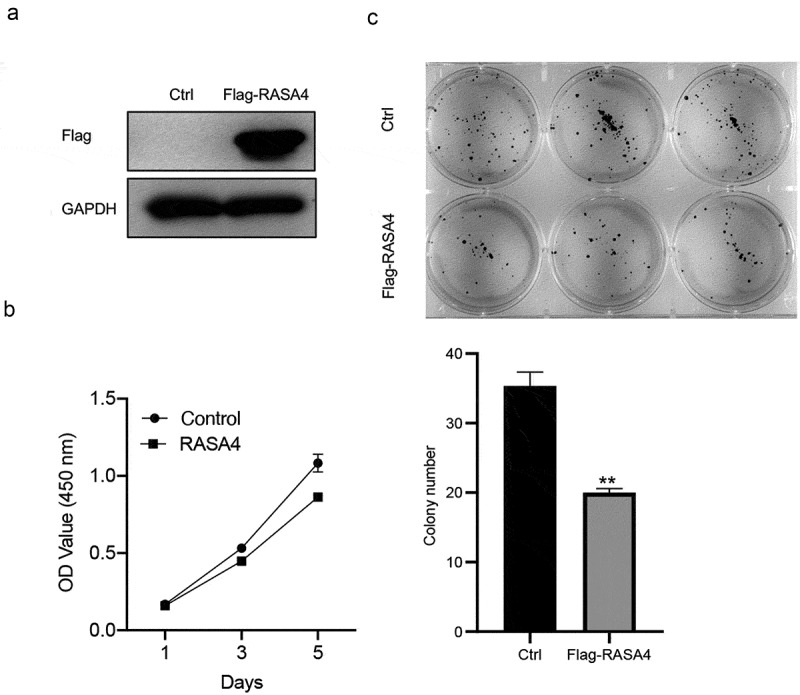
RASA4 overexpression suppresses proliferation and colony formation in HeLa cells. (a) Western blotting demonstrating transient RASA4 overexpression in HeLa cells. RASA4 was exogenously overexpressed through cell transfection with the pLV-Flag-RASA4 vector. (b) RASA4 overexpression weakened the proliferation of HeLa cells. Cell proliferation was assessed through CCK8 assays at the indicated timepoints. Data are shown as mean ± SEM (n = 3). (c) RASA4 overexpression impaired the colony generation rate of HeLa cells. Cell colony formation was measured through colony formation assays. Data are shown as mean ± SEM (n = 3)

To analyze the cellular function of RASA4 in the CESC tissue, we overexpressed RASA4 in HeLa cells through cell transfection with pLV-Flag-RASA4. Western blotting results demonstrated that the RASA4 expression was exogenously increased in HeLa cells when compared with that in cells transfected with the empty pLV-Flag vectors (Figure 2(a)). The CCK8 and colony formation assays were conducted to examine the consequence of RASA4 overexpression. The outcomes of CCK8 assays demonstrated that the proliferative capacities were weakened on the exogenously enforced expression of RASA4 in HeLa cells (Figure 2(b)). Similarly, the colony generation rate of HeLa cells was impaired after RASA4 overexpression (Figure 2(c)). Cumulatively, the enforced expression of RASA4 inhibited the proliferation and colony formation of HeLa cells *in vitro*.

### RASA4 depletion increases proliferation and colony formation in HeLa and C-33A cells

**Figure 3.**
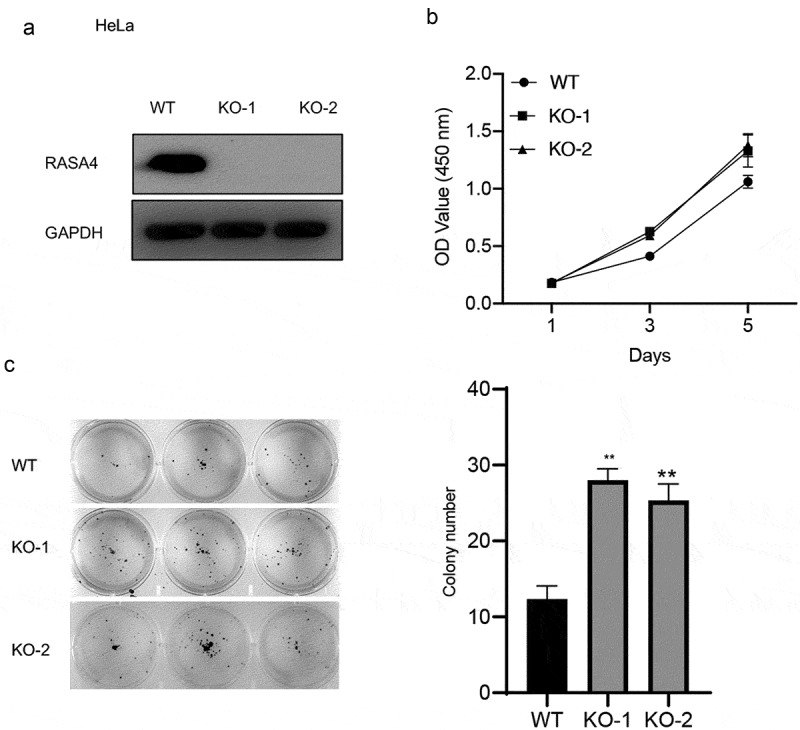
RASA4 depletion increases proliferation and colony formation in HeLa cells. (a) Western blotting demonstrated transient depletion of RASA4 in HeLa cells. RASA4 depletion was assessed using the CRISPR/Cas9 technology. (b) CCK8 assays demonstrated the impact of RASA4 deficiency on cell proliferation (n = 3). (c) Colony formation assays manifesting the impact of the RASA4 deficiency on the colony formation rate of HeLa cells

**Figure 4.**
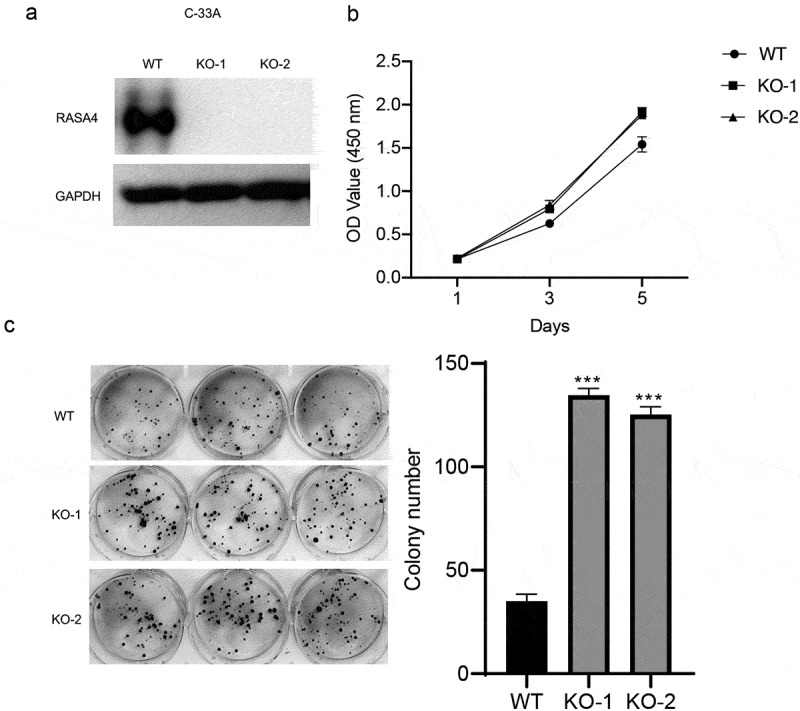
RASA4 depletion increases proliferation and colony formation in C-33A cells. (a) Western blotting demonstrated a transient depletion of RASA4 in the C-33A cells. RASA4 depletion was induced using the CRISPR/Cas9 technology. (b) CCK8 assays demonstrated the impact of RASA4 deficiency on cell proliferation (n = 3). (c) Colony formation assays manifested the impact of RASA4 deficiency on the colony formation rate of C-33A cells

To elucidate the role of RASA4 in CESC cells, we confirmed the effect of RASA4 on HeLa cell proliferation after complete depletion of RASA4 in cells by using the CRISPR/Cas9 technology. Western blotting results confirmed the genetic inactivation of RASA4 in HeLa cells (Figure 3(a)). Furthermore, the CCK8 assays manifested that the HeLa cell proliferation was increased by the loss of RASA4 (Figure 3(b)). A similar increase also was noted in the colony formation ability of the RASA4-deficient HeLa cells when compared with that of the untransfected cells (Figure 3(c)). Similarly, RASA4-deficient C-33A cells were established to further explore the role of RASA4 in cell proliferation (Figure 4(a)). Accordingly, elevated proliferation was observed in the C-33A cells when RASA4 was depleted (Figures 4(b,c)). Altogether, RASA4 deficiency resulted in the elevation of CC cell proliferation *in vitro*.

### RASA4 inactivates the HIF1α signaling pathway in CC

**Figure 5.**
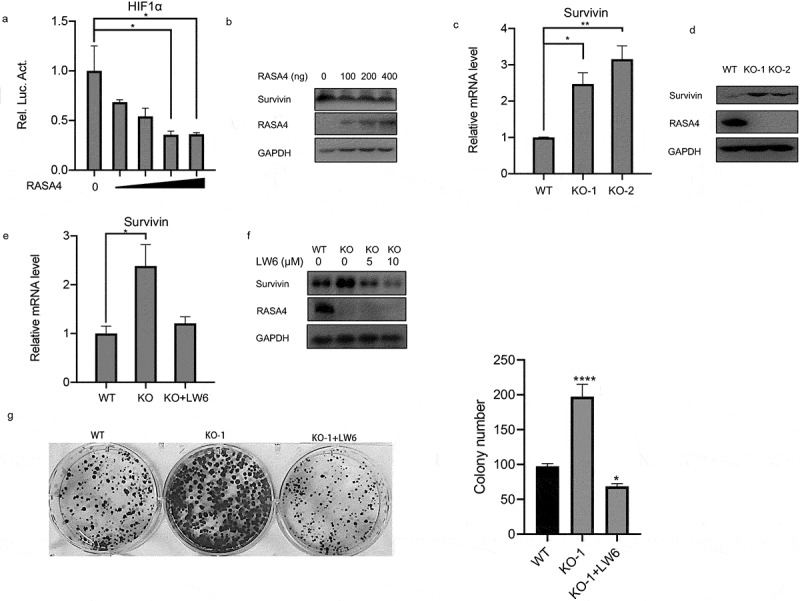
RASA4 inactivated the HIF1α signaling pathway in CESC. (a) Dual-luciferase reporter assay demonstrated that the HIF1α-mediated luciferase activities decreased in HeLa cells infected with different doses of pLV-Flag vectors (0, 100, 200, 400 ng). (b) Western blotting revealed that survivin protein was reduced in the HeLa cells infected with different doses of pLV-Flag vectors (0, 100, 200, and 400 ng). (c) Detection of the HIF1α-mediated luciferase activity in RASA4-deficient HeLa cells. (d) Western blotting revealed that the survivin protein expression was increased in HeLa cells when RASA4 depleted. (e) RT-qPCR results showed that the escalated survivin mRNA levels by RASA4 depletion were partly reversed by LW6 treatment. (f) Western blotting results unveiled a gradual decrement of survivin protein in the RASA4-overexpressing HeLa cells along with an increasing dose of LW6 treatment. (g) Colony formation assays demonstrated that the LW6 treatment could offset the robust escalation of the colony formation number in RASA4-deficient HeLa cells

It is well-established that different signaling events are involved in CC progression; therefore, we sought to investigate whether RASA4 suppression on HeLa cell proliferation is associated with the signaling pathway. Considering that both RASA4 and HIF1α signaling show a high relevance with the intracellular calcium levels [14], we adopted the dual-luciferase reporter assays to examine the change in the HIF1α signaling pathway when RASA4 overexpression or depletion occur to elucidate the potential mechanism of RASA4 in CC. As shown in Figure 5(a), the gradual deregulation of HIF1α-mediated luciferase activity was demonstrated in HeLa cells with a different dose of RASA4-overexpressing vectors. Survivin is known to be an essential activator in the HIF1α signaling pathway in CC cells. Consistently, Western blotting results revealed weakened survivin protein expression accompanied by the enforced expression of RASA4 in HeLa cells (Figure 5(b)). As expected, an increase in the survivin mRNA level was detected in RASA4-deficient HeLa cells (Figure 5(c)). Western blotting analysis revealed a marked increase in the expression of the survivin protein level in the HeLa cells when RASA4 depleted (Figure 5(d)). Therefore, we conducted rescued assays to verify whether RASA4 regulates proliferation through the HIF1α signaling pathway. For this purpose, we first treated the RASA4-deficient HeLa cells with 5-μM LW6. RT-qPCR results showed that LW6 treatment triggered an abrogation of robust survivin mRNA levels resulting from RASA4 deficiency (Figure 5(e)). Furthermore, we treated the RASA4-deficient HeLa cells with LW6 at different doses (0, 10, and 20 μM). The level of the survivin proteins decreased with an increasing dose of LW6 (Figure 5(f)). More importantly, the enhanced colony formation rate caused by RASA4 deficiency was hindered by LW6 treatment (Figure 5(g)). In summary, RASA4 could retard the proliferation of HeLa cells by inactivating the HIF1α signaling pathway.

### RASA4 depletion favors xenograft tumor growth

**Figure 6.**
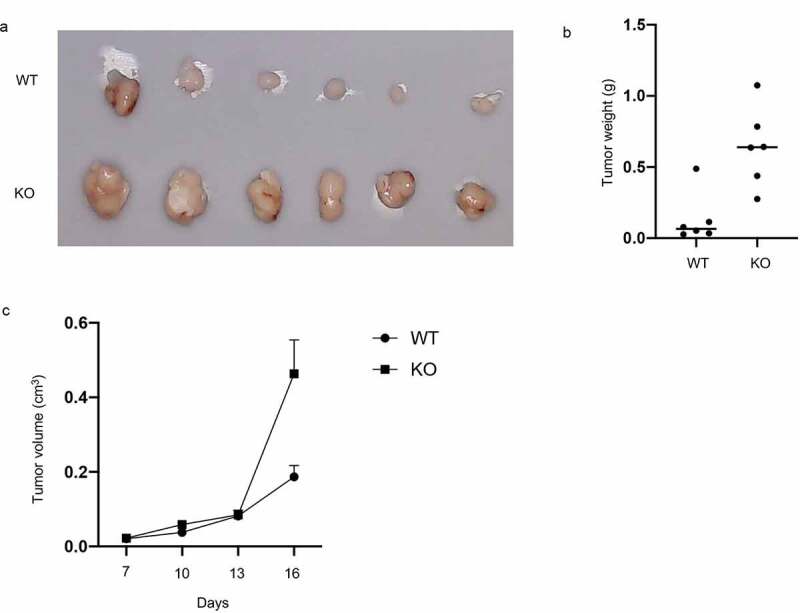
Knockout of RASA4 favors the growth of xenograft tumor in nude mice. (a) Pictures of tumor grown under RASA4 depletion; (b) The tumor volume growth curves from day 0 to day 16; (c) Tumor weight was analyzed after sacrificing the mice

Because of a strong inhibitory effect of RASA4 on CC growth *in vitro*, we further detected its effect *in vivo*. BALB/c mice were pre-treated subcutaneously with the RASA4-deficient C-33A cells to establish a human CC xenograft tumor model. The tumor weight and size were detected subsequently. As shown in Figure 6, the mice bearing RASA4-deficient C-33A tumor displayed a significantly enhanced xenograft tumor relative to the control group (Figures 6(a,b)). Finally, tumor weights were determined after the mice were sacrificed. As depicted in Figure 6(c), the RASA4-deficient C-33A group also manifested tumor progression.

## Discussion

In the present study, through bioinformatics analysis, we demonstrated that the CC tissues displayed lower RASA4 mRNA levels than the noncancerous cervical tissues. Cell functional assays for RASA4 overexpression showed a reduced proliferative potency of HeLa cells, whereas RASA4 depletion increased CC growth *in vitro* and *in vivo*. Mechanically, RASA4 overexpression conspicuously diminished the HIF1α signaling pathway in HeLa cells. The *in vitro* application of a specific inhibitor of HIF1α revealed that HIF1α was implicated in RASA4-regulated proliferation of HeLa cells. Our findings provide novel evidence for the role of RASA4 in the malignancy of CC by the HIF1α signaling pathway.

Ras and its associated proteins were frequently reduced in various human malignancies because constitutive hyperactivation of Ras mutant activities resulted in uncontrolled cellar behaviors and association with cellar transformation, eventually leading to human malignancies [15–17]. RASA4 acts as a Ras GTPase-activating protein, which belongs to the RASA family. In addition to RASA4, this family comprises RASA1, RASA2, RASA3, and RASA5. Studies have reported the function of the RASA family members in different cancers. For example, RASA5 could counteract the oncogenic response in different cancer cells resulting from the activation of RAS signaling and then serves as a tumor suppressor [9]. RASA3 is poorly expressed in hepatocellar carcinoma in association with promoter hypomethylation, which is associated with the clinical features of tumor patients [18]. Furthermore, the inactivation of RASA2 activates the RAS signaling and favor the malignant phenotypes of melanoma cells [19]. A recent investigation on resistant juvenile myelomonocytic leukemia revealed RASA4 deregulation in cancer tissues and dismal prognosis [11]. In line with previous investigations, we found that the RASA4 mRNA level was reduced in the CC tissues. Poor expression of RASA4 is related to poor prognosis. Meanwhile, RASA4 expression signified promising dialogistic values to differentiate the CC patients from healthy individuals. Taken together, these results indicate the tumor-suppressing role of RASA4 in CC malignancy. Further *in vitro* loss – and gain- functional assays were performed to confirm that RASA4 could impair the proliferative potential of HeLa and C-33A cells. The *in vitro* results were confirmed through *in vivo* assays. Therefore, our results strongly suggest that RASA4 acts as a tumor suppressor in CC progression.

Prognostic evaluations have underscored the prognostic power of minimal intratumoral oxygen partial pressure or hypoxia in CC [20]. As a transcriptional factor by hypoxia, HIF1α actively participates in the regulation of a complex transcription regulatory network central to tumor growth, enabling CC progression. For example, in CC cells, HIF1α increases survivin expression, which favors tumor cell invasion and dissemination and metastasis [21]. HIF-1α activates vascular endothelial growth factor signaling circuits, thereby boosting the malignant behaviors of CC cells [22]. In addition, HIF1α is highly expressed in CC and is evidently correlated with the clinicopathological characteristics [21]; its gene polymorphisms are thus an influential risk factor for patients with CC [23]. Considering a possible negative response to the intracellular calcium ion level between the RASA4 and HIF1α signaling pathway [14], we further investigated the impact of RASA4 on the HIF1α signaling pathway. Herein, for the first time, we recorded that RASA4 overexpression can significantly and dose-dependently reduce the HIF1α transcriptional activity and survivin expression. Furthermore, the HIF1α transcriptional activity and survivin expression were obviously reduced in the RASA4-knockout HeLa cells. More importantly, LW6 treatment not only abrogated the increased survivin expression in HeLa cells but also reversed the increased clonogenic activities of these cells by causing RASA4 depletion. These findings suggested that RASA4 could inhibit the proliferation of HeLa cells partially by inactivating the HIF1α signaling. RAS has been reported to bind to HIF-1α. Our present results are thus consistent with previous findings suggesting that RASA4 may cooperatively interact with RAS and HIF-1α to inhibit HeLa cell proliferation and abrogate the cytotoxic T-lymphocytes’ ability to kill tumor cells [24].

## Conclusion

The study revealed the downregulation of the RASA4 mRNA levels in the CC tissues. The *in vitro* functional assays demonstrated that RASA4 could suppress proliferation through deregulation of the HIF1α signaling pathway. Our investigation underscored the importance of the RASA4/HIF1α axis in CC progression. Our findings thus offer novel mechanistic insights into CC progression and propose an effective candidate to target the underlying mechanisms to control proliferation.
